# Novel Potential Janus Kinase Inhibitors with Therapeutic Prospects in Rheumatoid Arthritis Addressed by In Silico Studies

**DOI:** 10.3390/molecules28124699

**Published:** 2023-06-12

**Authors:** Andrei-Flavius Radu, Simona Gabriela Bungau, Andrei Paul Negru, Bogdan Uivaraseanu, Mihaela Alexandra Bogdan

**Affiliations:** 1 Doctoral School of Biological and Biomedical Sciences, University of Oradea, 410087 Oradea, Romania; andreiflavius.radu@uoradea.ro (A.-F.R.); uivaraseanu_bogdan@yahoo.com (B.U.); 2 Department of Preclinical Disciplines, Faculty of Medicine and Pharmacy, University of Oradea, 410073 Oradea, Romania; 3 Department of Pharmacy, Faculty of Medicine and Pharmacy, University of Oradea, 410028 Oradea, Romania; mihaela.alexandra.bogdan@gmail.com

**Keywords:** rheumatoid arthritis, Janus kinase inhibitors, DMARDs, protein targets, molecular docking, virtual screening

## Abstract

Rheumatoid arthritis (RA) is a debilitating autoimmune disorder with an inflammatory condition targeting the joints that affects millions of patients worldwide. Several unmet needs still need to be addressed despite recent improvements in the management of RA. Although current RA therapies can diminish inflammation and alleviate symptoms, many patients remain unresponsive or experience flare-ups of their ailment. The present study aims to address these unmet needs through in silico research, with a focus on the identification of novel, potentially active molecules. Therefore, a molecular docking analysis has been conducted using AutoDockTools 1.5.7 on Janus kinase (JAK) inhibitors that are either approved for RA or in advanced phases of research. The binding affinities of these small molecules against JAK1, JAK2, and JAK3, which are target proteins implicated in the pathophysiology of RA, have been assessed. Subsequent to identifying the ligands with the highest affinity for these target proteins, a ligand-based virtual screening was performed utilizing SwissSimilarity, starting with the chemical structures of the previously identified small molecules. ZINC252492504 had the highest binding affinity (−9.0 kcal/mol) for JAK1, followed by ZINC72147089 (−8.6 kcal/mol) for JAK2, and ZINC72135158 (−8.6 kcal/mol) for JAK3. Using SwissADME, an in silico pharmacokinetic evaluation showed that oral administration of the three small molecules may be feasible. Based on the preliminary results of the present study, additional extensive research is required for the most promising candidates to be conducted so their efficacy and safety profiles can be thoroughly characterized, and they can become medium- and long-term pharmacotherapeutic solutions for the treatment of RA.

## 1. Introduction

Rheumatoid arthritis (RA) is a chronic, inflammatory disorder with major implications for the immune system that predominantly harms the synovial joint lining and is correlated with financial burdens, premature mortality, and gradual disability [1]. Furthermore, it impacts women more often than men and is most frequently identified in elderly people [2]. The first 3 months after the onset of initial symptoms are assumed to represent the appropriate therapeutic window, so early diagnosis is acknowledged as the main strategic determinant for achieving the most beneficial results, including limited joint damage, no functional impairment, and minimal radiologic development [3].

The associations between genetic factors (i.e., human leukocyte antigen D-related alleles, tumor necrosis factor-receptor associated factor 1, and complement component 5 tyrosine phosphatase non-receptor type 22 related genes, etc.) and environmental factors (i.e., silica dust exposure, smoking, reduced ultraviolet B light, infections, etc.) are the most significant risk factors with implications for the development of RA [4]. 

Various mechanistic concepts have been proposed, even though the pathophysiological processes underlying RA are still not completely understood. According to experimental assessments, autoimmune responses might actually occur years before manifestations of joint inflammation become noticeable, referred to as the pre-RA phase [5]. Citrullinated proteins (i.e., vimentin, type II collagen, fibronectin, etc.) are no longer detected as self-structures by the immune system due to the implications of human leukocyte antigen-DR1 and human leukocyte antigen-DR4 susceptibility genes [6]. 

Multiple pathways are activated, and a variety of proteins are involved throughout the pathophysiological mechanisms of RA, including cytokines, adhesion molecules, matrix metalloproteinases, interferon signaling, receptor activators of nuclear factor kappa B ligand, B cells, etc. [7,8]. 

Among the most relevant molecular targets are Janus kinases (JAKs), a family of intracellular enzymes that have the potential to function as therapeutic targets due to the fact that they contribute significantly to controlling the JAK-signal transducer and activator of transcription proteins pathway, which regulates signal transduction pathways associated with inflammation and immune responses [9,10].

JAK1, JAK2, JAK3, and tyrosine kinase 2 (TYK2) constitute the JAK family. Multiple studies have revealed that synovial cells and synovial tissue express various JAK isoforms as well as downstream signal transducers and activators of transcription proteins [9,11]. 

Pharmacological approaches to investigating numerous potential molecules that could improve the current treatment for RA have advanced significantly because of continuous development in the strategies and methods used in drug design processes. According to the European League Against Rheumatism and American College of Rheumatology recommendations, available treatments address RA from two viewpoints: management of the symptoms by using nonsteroidal anti-inflammatory drugs [12] and glucocorticoids, and disease-modifying management by utilizing disease-modifying antirheumatic drugs (DMARDs) [13,14].

Conventional synthetic DMARDs (csDMARDs), biologic DMARDs (bDMARDs), and targeted synthetic DMARDs (tsDMARDs) are various types of designed DMARDs [15]. For recently diagnosed RA patients, csDMARDs are frequently utilized as the initial line of treatment. When the first-line treatment is not tolerated or is inefficient, bDMARDs or tsDMARDs are indicated. tsDMARDs are orally active, small compounds that penetrate the cytoplasm and directly affect intracellular signaling by inhibiting kinases or phosphodiesterases, as opposed to bDMARDs, which are large molecules that require parenteral administration [16]. 

Five JAKis (tofacitinib, baricitinib, peficitinib, upadacitinib, and filgotinib) have currently been authorized by several agencies for the treatment of RA and are considered to be tsDMARDs. All the JAKis that are officially approved are competitive antagonists. Reportedly, it is considered that tofacitinib inhibits JAK1, JAK2, and JAK3 to variable degrees in vivo [9]. Upadacitinib and filgotinib are both selective JAK1 inhibitors, whereas baricitinib inhibits both JAK1 and JAK2 [17]. When tested against JAK3 and JAK1, peficitinib was 14 times more effective than JAK2 [18].

Other JAKis, such as VX-509 (decernotinib), ruxolitinib (INCB018424), and itacitinib, are in various phases of clinical trials evaluating their potential in the treatment of RA. Decernotinib has been synthesized as a selective JAK3 inhibitor and has been observed to have a reduced action on other JAKs, ruxolitinib inhibits JAK1 and JAK2 to a greater extent, while itacitinib selectively inhibits JAK1 [10].

**Figure 1:**
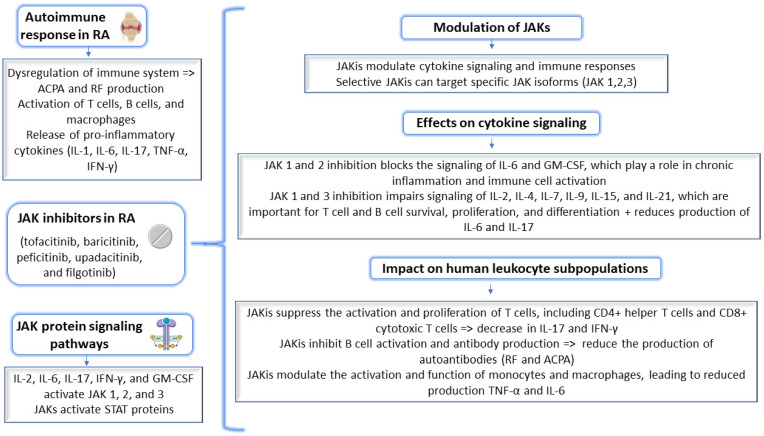
Targeting cytokine signaling in RA by JAKis and modulating the autoimmune response. RA, rheumatoid arthritis; ACPA, anti-citrullinated protein antibodies; RF, rheumatoid factor; IL, interleukin; TNF-α, tumor necrosis factor alpha; JAK, Janus kinse; JAKis, Janus kinase inhibitors; IFN-γ, interferon gamma; GM-CSF, granulocyte-macrophage colony-stimulating factor; and STAT, signal transducer and activator of transcription protein.

To improve the medical applications of JAKis, especially if they are considered in the context of cytokine signaling, it is crucial to comprehend how they can modulate or ameliorate the autoimmune response in RA. Figure 1 shows the molecular-level implications of JAKis in the context of autoimmune mechanisms in RA.

By targeting JAK signaling and modulating cytokine responses in human leukocyte subpopulations, JAKis reduced inflammation, attenuated joint injury, and alleviated the signs and symptoms of RA, according to experimental studies [19,20]. These effects reduce the immune response and provide beneficial treatments for RA patients.

Tofacitinib was designed to attach to the ATP-binding site of JAK3 in a competitive manner. Initially believed to inhibit JAK3 phosphorylation only, tofacitinib is now shown to inhibit JAK1, JAK2, and JAK3 to variable degrees in vitro and in vivo. Tofacitinib is an effective inhibitor of JAK1 and JAK3, but it is less effective against JAK2 and TYK2, according to in vitro kinase studies [21]. In addition, baricitinib is a selective inhibitor of JAK1 and JAK2, and upadacitinib and filgotinib are selective inhibitors of JAK1 [19,22,23].

In an experimental study, the in vitro effect of tofacitinib was compared to that of other JAKis using healthy donors’ peripheral blood mononuclear cells. The investigators measured the levels of phosphorylated signal transducer and activator of transcription (STAT) in the presence of various JAKis following cytokine stimulation. This allowed them to evaluate the effect of each JAKi on cytokine signaling pathways. In addition, the researchers calculated the half-maximal inhibitory concentration (IC50) values for each JAKi in distinct leukocyte subpopulations. By comparing the calculated mean concentration–time profiles of JAKi-treated subjects over a period of 24 h, the therapeutic significance of the in vitro analysis was determined. For each JAKi, cytokine, and cell type, they determined the duration above IC50 and the average daily percent inhibition of pSTAT formation. The results demonstrated that various JAKis had distinct in vitro pharmacological profiles. Tofacitinib and upadacitinib were the most potent inhibitors of IL-2, IL-4, IL-15, and IL-21, which are JAK1- and 3-dependent cytokines. They had lower IC50 values and longer times above IC50, indicating a higher inhibition of STAT signaling over the course of the dosing interval [19].

Variations in biomarkers of inflammation, levels of cytokines, immune cell populations, or specific proteins associated with the illness pathway can be used to measure the in vivo effect of tofacitinib. Single-dose tofacitinib data in a collagen-induced arthritis mouse model revealed immediate effects of tofacitinib on plasma inflammatory mediators and, in some cases, a sustained impact on chemotactic proteins, but no immediate impact on the disease severity index. It has been demonstrated that IL-6 and interferon gamma-inducible protein-10 can be inhibited rapidly. Other mechanistic assessments have shown that tofacitinib inhibition of cytokine receptor signaling in T cells can ultimately result in suppression of IL-12 as well as decreased production of inflammatory T helper 17 cells generated in response to IL-6 and IL-23 [24]. In addition, the in vivo effect of tofacitinib can also be evaluated in clinical trials where changes in disease activity parameters are measured. Tofacitinib decreased the median disease activity score (DAS28) in the presence of csDMARDs from 4.4 to 2.6 [25].

JAKis have emerged as a significant therapeutic approach for RA patients and other inflammatory disorders [26]. Despite JAKi’s efficacy, there continue to be several unmet needs in its development and application. It is necessary to provide more affordable therapy options because the cost of JAKis might be excessive for many RA patients [27]. JAKis’ long-term safety is still under debate, especially in light of the possibility of infections [28], higher cholesterol levels [29], and the risk of developing various malignancies [30]. However, improvements in efficacy are identified as among the most important unmet needs. While JAKis have been shown to be beneficial in alleviating RA symptoms and slowing the progression of joint degeneration, a significant number of patients remain unresponsive or experience flare-ups of their condition [31]. 

Although valuable, current RA therapeutic management has limitations. Therefore, more advanced treatments are required for addressing specific unmet needs. The latest research is aimed at developing novel and improved JAKis [10,32], improving patient selection and dosage approaches, and evaluating combination therapies to address these unmet needs [9,33].

Molecular docking studies are valuable because they can provide insights into the potential binding affinity of small drug molecules to JAK proteins with implications for RA, but experimental endorsement is needed to validate the results and determine their genuine therapeutic potential as RA therapies. By modulating the interaction between the drug candidate and the JAK target protein, compounds with strong binding affinities and specificities for JAK can be identified, making them potential candidates for further investigation. Furthermore, the medical applicability of these studies in their final form is found in the design of novel JAKis that are efficient, secure, and cost-effective, offering patients with RA better therapy alternatives [32].

Therefore, the aim of this study was to apply molecular docking, virtual screening, and pharmacokinetic in silico strategies for identifying compounds with the highest affinity to the target protein (JAKs), converting compounds not used in current medical practice into substances with potential use in the management of RA. The contribution made to the field of study emerges from the need to find and establish novel compounds with therapeutic action in RA to address the current limitations, with the present study addressing the possible interactions with JAK1, JAK2, and JAK3 through a distinct design.

## 2. Results and Discussion

### 2.1. Molecular Docking Approach Targeting JAK1

The grid box size was set to 60 × 60 × 60 Å and its coordinates were set to X = 10.79, Y = 13.83, and Z = −15.33. The Protein Data Bank (PDB) ID of JAK1 is 3EYG.

The calculated root-mean-square deviation (RMSD) value (1.371 Å) fit within the accepted literature values (<2 Å), indicating that the structure of the docked tofacitinib overlapped to a large extent with the structure of the co-crystallized tofacitinib and that the docking method adequately simulates the ligand–protein interaction.

**Figure 2:**
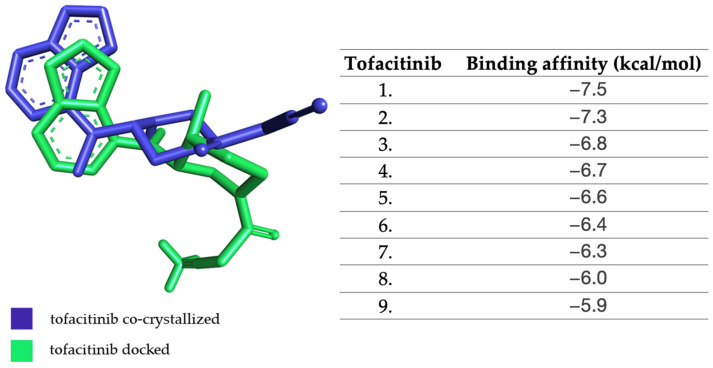
The highest binding affinity values for JAK1 of the most stable conformations of tofacitinib.

Following the docking process, the co-crystallized tofacitinib in its best conformation showed an affinity of −7.5 kcal/mol towards JAK1. In Figure 2, the structure of the docked tofacitinib is overlaid with the structure of the co-crystallized tofacitinib, and the affinities of the most stable conformations of tofacitinib towards JAK1 are depicted.

The binding affinities of the ligands studied for their potential to interact with JAK1 are shown in Table 1.

It has been shown that itacitinib has the highest binding affinity for JAK1 of all the ligands studied, signifying that itacitinib has the highest potential for a strong interaction with JAK1, which may lead to its inhibition and subsequent expression of its therapeutic effects. 

Filgotinib, with a docking score of −9.1 kcal/mol, has the highest affinity for JAK1 of the JAKis approved by regulatory agencies for the treatment of RA. 

**Figure 3:**
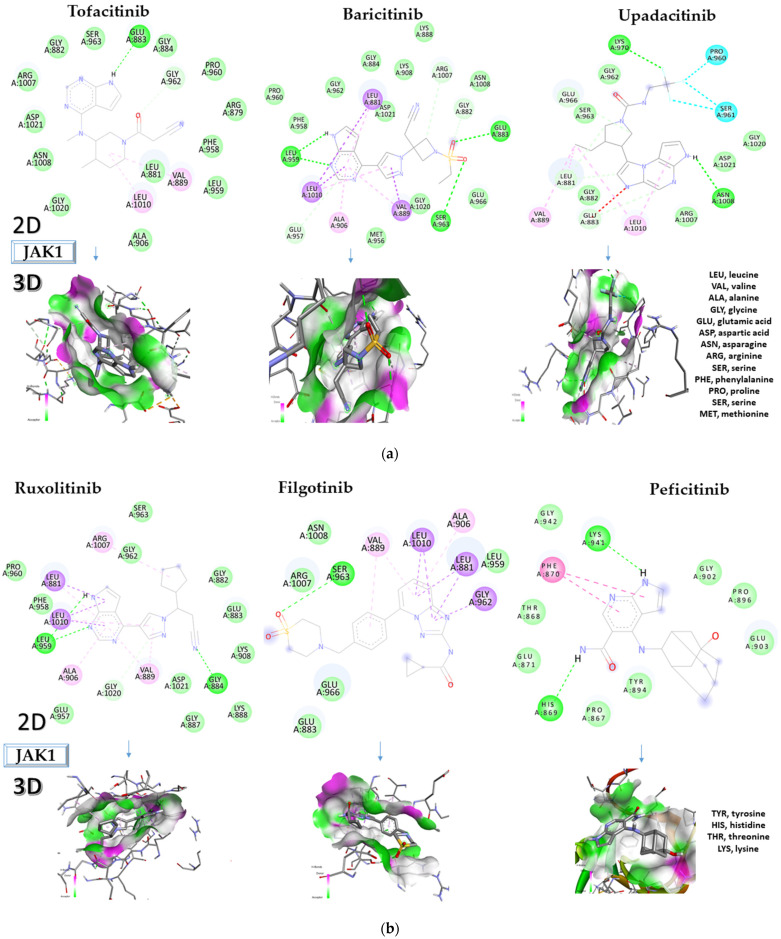

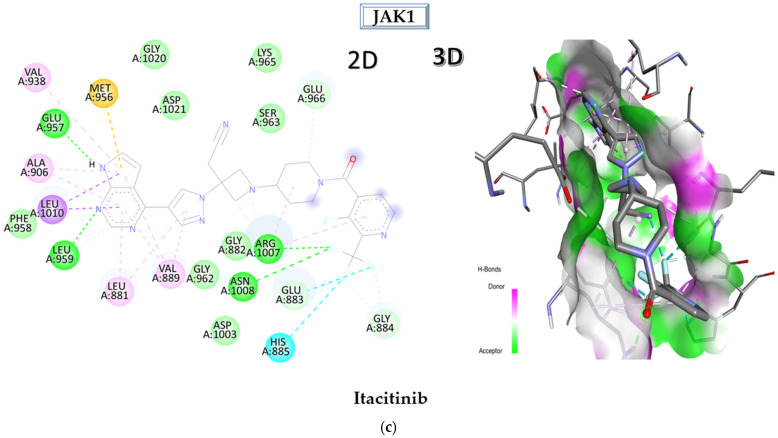
Interactions between some amino acids of JAK1 and the compounds tested in 2D and 3D formats. JAK1, Janus kinase 1. (**a**) Tofacitinib, baricitinib, and upadacitinib interactions with JAK1; (**b**) ruxolitinib, filgotinib, and peficitinib interactions with JAK1; and (**c**) itacitinib interactions with JAK 1.

The type and potency of interactions between JAKis and amino acids in JAK1 determines the JAKi’s binding affinity for JAK1. The JAKis’ effectiveness and specificity for JAK1 are significantly influenced by the particular amino acids that they interact with in JAK1. Figure 3a–c depicts the in-depth 2D and 3D interactions of ligands with different amino acids of JAK1.

Following the analysis of the 2D diagrams, it has been observed that the control ligand, tofacitinib, interacts with GLU883, VAL889, and LEU1010.

Baricitinib interacts with LEU881, LEU959, LEU1010, GLU957, ALA906, VAL889, SER963, GLU883, GLY882, and ARG1007, and upadacitinib, having the lowest docking score (−6.4 kcal/mol), interacts with SER961, PRO960, LYS970, GLU966, LEU881, VAL889, GLU883, LEU1010, and ASN1008. 

Ruxolitinib has a binding affinity of −9.0 kcal/mol and binds to the following amino acids: GLY962, ARG1007, LEU881, LEU1010, LEU959, ALA906, GLY1020, VAL889, and GLY884.

Peficitinib has an affinity of −6.5 kcal/mol towards JAK1 and interacts with the following amino acids: LYS941, PHE870, and HIS869. Filgotinib showed an affinity of −9.1 kcal/mol and interacted with the following amino acids: GLY962, LEU881, ALA906, LEU1010, VAL889, and SER963.

Itacitinib, the compound showing the highest affinity for JAK1 (−9.7 kcal/mol), binds to the following amino acids: MET956, VAL938, GLU957, ALA906, LEU1010, LEU959, LEU881, VAL889, ARG1007, ASN1008, GLU883, HIS885, GLY884, and GLU966.

The increase in the number of interactions between the amino acids of JAK1 protein and the tested compounds may be associated with higher binding affinities and greater potential for JAK1 inhibition, but this hypothesis needs to be confirmed by further research.

### 2.2. Molecular Docking Approach Targeting JAK2

Similar to the protocol for JAK1, the native PDB protein ligand with ID 6VNE was redocked, and the RMSD was calculated. In the case of JAK2, the protein was co-crystallized with fedratinib. The calculated RMSD value for docked versus co-crystallized fedratinib is 1.271 Å. The size of the grid box is unchanged (60 × 60 × 60 Å) and the coordinates are X = 36.19, Y = −1.51, and Z = 10.55.

**Figure 4:**
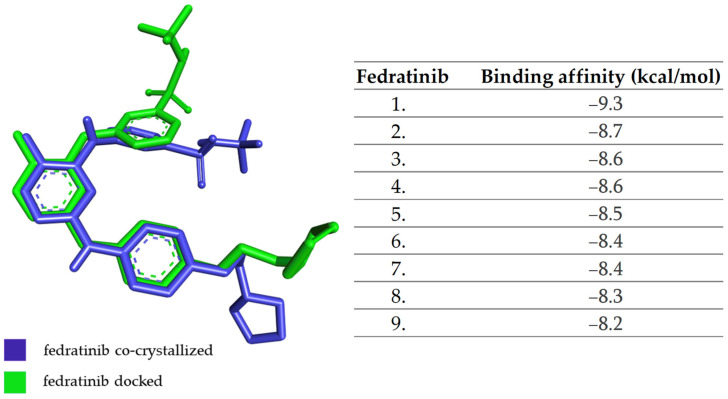
The highest binding affinity values for JAK2 of the most stable conformations of fedratinib.

In Figure 4, the structure of co-crystallized fedratinib has been overlaid with that of docked fedratinib, and the affinities of the most stable conformations of fedratinib towards JAK2 are depicted.

Subsequently, other ligands, including baricitinib [19], ruxolitinib [10], and peficitinib [18], all with demonstrated affinities for JAK2, have been docked. Protein preparation was carried out according to the data presented in the materials and methods section. The binding affinities of the docked ligands are presented in Table 2.

Of the examined ligands, peficitinib has the highest affinity for JAK2, with −9.5 kcal/mol, and interacts with the following amino acids: ARG980, GLY993, LEU983, ALA880, LEU932, LEU855, and VAL863. Ruxolitinib, which has a lower affinity than peficitinib (−8.5 kcal/mol) interacts with ASP994, ASN981, ARG980, ALA880, LEU983, LEU855, and VAL863, while baricitinib has an affinity of −8.3 kcal/mol and interacts with GLY858, SER936, VAL863, LEU855, MET929, GLU930, ALA880, LEU932, and LEU983. 

**Figure 5:**
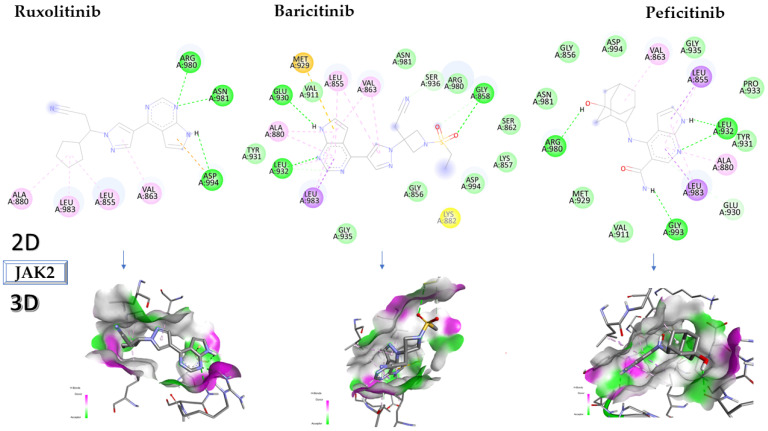
Interactions between some amino acids of JAK 2 proteins and compounds tested in 2D and 3D formats. JAK2, Janus kinase 2.

Figure 5 depicts the in-depth 2D and 3D interactions of ligands with different amino acids of the JAK1 protein.

### 2.3. Molecular Docking Approach Targeting JAK3

The target protein (PDB ID: 5TTV) was co-crystallized with the compound entitled covalent inhibitor 6. The grid box size remained 60 × 60 × 60 Å, as with the other proteins tested, and its coordinates were set to X = −0.04, Y = 17.28, and Z = −5.04. The RMSD value of the co-crystallized covalent inhibitor 6 versus the docked one is 1.634. 

**Figure 6:**
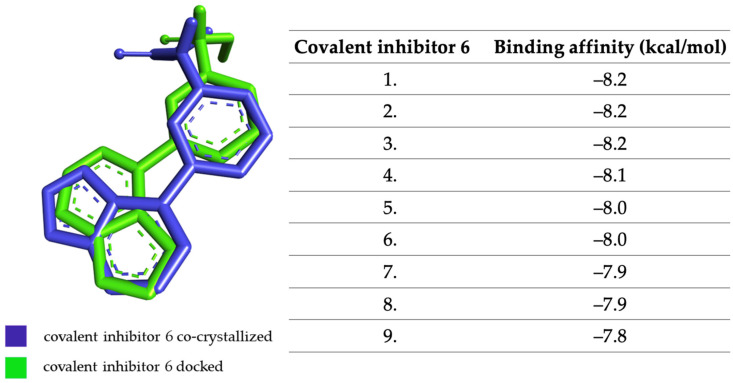
The highest binding affinity values for JAK3 of the most stable conformations covalent inhibitor 6.

In Figure 6, the structure of the co-crystallized inhibitor 6 structure has been superimposed on that of the docked structure, and the affinities of the most stable conformations of the covalent inhibitor 6 towards JAK3 are shown.

Ligands with demonstrated high affinity for JAK3 and potential in RA therapy are tofacitinib [19], decernotinib [34], and peficitinib [18]. For these compounds, the binding affinity towards JAK3 is calculated, and the results are shown in Table 3.

Decernotinib interacts with the following amino acids: CYS909, LEU828; MET902; LEU905; LEU956; ALA853; GLU903; VAL884; and VAL836.

Tofacitinib interacts with MET902, VAL836, LEU956, ALA828, ALA853, GLU903, LEU905, and VAL884; peficitinib, the ligand with the highest affinity for JAK3, interacts with LEU905, LEU828 ALA853, LEU956, GLU903, MET902, and VAL836.

**Figure 7:**
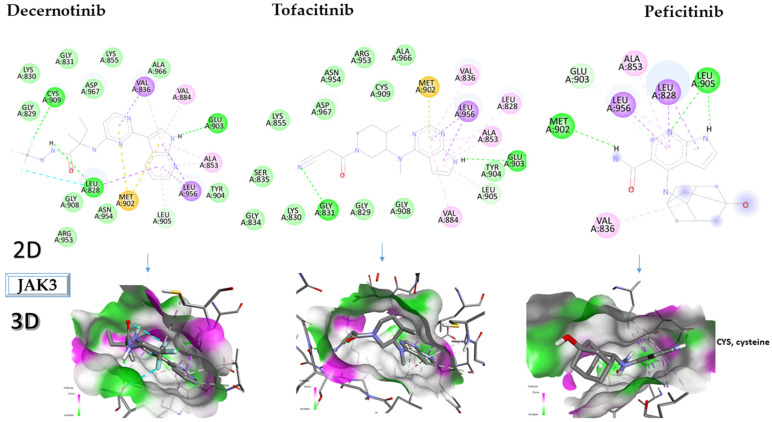
Interactions between some amino acids of the JAK3 protein and compounds tested in 2D and 3D formats. JAK3, Janus kinase 3.

Figure 7 presents the in-depth 2D and 3D interactions of ligands with different amino acids of the JAK3 protein.

### 2.4. Virtual Screening of the Most Promising Compounds

In order to identify new compounds with potential use in the treatment of RA, the data obtained from the molecular docking study on the chemical structure of the compounds that showed the highest affinity for JAK1, JAK2, and JAK3 were processed. By inputting and rendering the data into the SwissSimilarity online platform (http://www.swisssimilarity.ch/, accessed on 22 March 2023), ligand-based virtual screening was performed to identify compounds with similar chemical structures, thus presenting potential for use in the management of RA, but requiring confirmation based on in-depth studies.

SwissSimilarity is an online application that offers an accessible platform for ligand-based virtual screening of chemical libraries in order to discover molecules that are similar to a query structure. SwissSimilarity provides multiple compound databases for screening, including authorized pharmaceuticals, widely accessible commercial compounds, and acknowledged biomolecules [35]. According to the extended-connectivity fingerprints (ECFP) approach, the chemical structure is “split” into many substructures that are identified by circular atom neighbors. These substructures are given integer identifiers, which are then mapped via a hashing method onto a binary vector of the required length. High detail encoding of a specific chemical structure is possible using ECFP, which also consistently performed well in comparative experiments [36].

A search for target compounds in the ZINC online database (https://zinc.docking.org/, accessed on 5 April 2023) was performed using the ECFP.

Based on the structure of itacinib, the compound with the highest binding affinity to JAK1, 36 structures were found with similarity scores ranging from 0.442 to 0.301. The similarity score shows the similarity between itacitinib and the newly identified compounds. The name assigned by the ZINC database, the chemical structure in 2D format, and the protein affinity resulting from the docking process for the five most relevant (highest binding affinities to JAK1) compounds not used in medical practice are shown in Table 4. 

The compound ZINC000252492504 presented the highest binding affinity for JAK1 in the present virtual screening study, suggesting that it may be a potential candidate for developing a drug to target JAK1. The binding affinity quantifies the interaction intensity between a ligand and its target protein. It assesses the ligand–protein complex’s stability, and a more negative value suggests a greater affinity for binding. The scientific literature contains values ranging from −6.28 kcal/mol to −18.70 kcal/mol for compounds with known action against JAK1 and those investigated for their potential impact on this protein. This range encompasses the binding affinity of ZINC000252492504 (i.e., −9.0 kcal/mol). Comparing ZINC000252492504 favorably with reported values suggests that its binding affinity is consistent with that of compounds known to target JAK1 [37,38].

Like the protocol for JAK1, molecular docking evaluation suggested that peficitinib (9.5 kcal/mol) had the highest affinity for the amino acids of JAK2 protein. Based on the structure of peficitinib, 29 ligands were identified. The name assigned by the ZINC database, the 2D structure, the similarity score, and the affinity towards JAK2 resulting from the docking process for the five most promising compounds not used in pharmacotherapies are shown in Table 5. 

The compound ZINC000072147089 presented the highest binding affinity for JAK2 in the present virtual screening study, suggesting that it may be a potential candidate for developing a drug to target JAK2 due to a possible effective interaction with the target. The binding affinities of compounds targeting JAK2 have been reported in the literature from in silico studies to range from −2.93 kcal/mol to −10.13 kcal/mol. Furthermore, drugs with known activity show a binding affinity typically starting at −6.84 kcal/mol [39,40]. In the present analysis, the binding affinity of ZINC000072147089 (i.e., −8.6 kcal/mol) falls within the range commonly found in the scientific literature.

Peficitinib also showed the highest affinity for JAK3. Based on the structure of peficitinib, 29 ligands with structural similarities were identified in the platform. The name assigned by the ZINC database, the 2D structure, the similarity score, and the affinity towards JAK3 resulting from the docking process for the five most promising compounds not used in pharmacotherapies are shown in Table 6.

In the scientific literature, the reported binding affinities of compounds targeting JAK3, as determined through in silico studies, range from −5.64 kcal/mol to −9.97 kcal/mol [39,41]. In the present analysis, the binding affinity of ZINC000072135158 towards JAK3 was determined to be −8.6 kcal/mol. This value falls within the range commonly observed in the scientific literature for compounds targeting JAK3. The similarity of the binding affinity of ZINC000072135158 to other compounds investigated for their interaction with JAK3 suggests that it may have the potential to effectively interact with and modulate the activity of JAK3.

Based on substances with pharmacological activity on JAK1, JAK2, and JAK3 demonstrated in the medical literature, itacinib and peficitinib showed the highest binding affinities for JAK isoforms. Structural similarity studies obtained from the assays identified 65 compounds whose affinity for target proteins was tested. 

The compound ZINC000252492504 showed the highest affinity for JAK1 among the ligands tested. From the 2D analysis, it has been identified that it interacts with GLU883, VAL889, LEU1010, PHE958, ALA906, LEU959, MET956, ALA881, ARG1007, GLY882, and GLU966. The 6,7-dihydro-5H-pyrazolo[5,1-b][1,3]oxazine group interacts with the following amino acids: GLU883 and GLU966. The piperidine group is involved in interaction with GLU966, GLY882, and ARG107. The piperazine group only interacts with LEU881. Most of the interactions between the molecule and the protein occur through the pyridine group and the fluorine atoms, which are frequently used to improve the properties of chemical compounds. These functional groups interact with the following amino acids: LEU881, MET956, LEU959, ALA906, PHE958, LEU1010, and VAL889.

For JAK2, the compound showing the highest affinity is ZINC000072147089, which binds to the following amino acids: LEU855, ALA880, LEU983, TYR931, GLU930, and LEU932. The cyclopentane group present in the molecule does not contribute to any interactions with the protein. On the other hand, the 1H-pyrrolo[2,3-b]pyridine group exhibits interactions with the amino acid LEU855. LEU855, ALA880, LEU983, TYR931, GLU930, and LEU932 interact with the protein through the fluorobenzamide group. 

In the case of JAK3, ZINC000072135158 shows the highest affinity and binds to the following amino acids: LEU956, LEU828, ALA853, LYS855, ASP967, VAL836, and CYS909. The interaction of the compound with CYS909 is facilitated by the presence of a cyclopentane group. Additionally, the molecule has the ability to interact with LEU828, LEU956, and ALA853 due to the presence of a 1H-pyrrolo[2,3-b]pyridine ring. Furthermore, interaction with ASP967, VAL836, and LYS855 is made possible by the 3-pyridinecarboxamide group.

**Figure 8:**
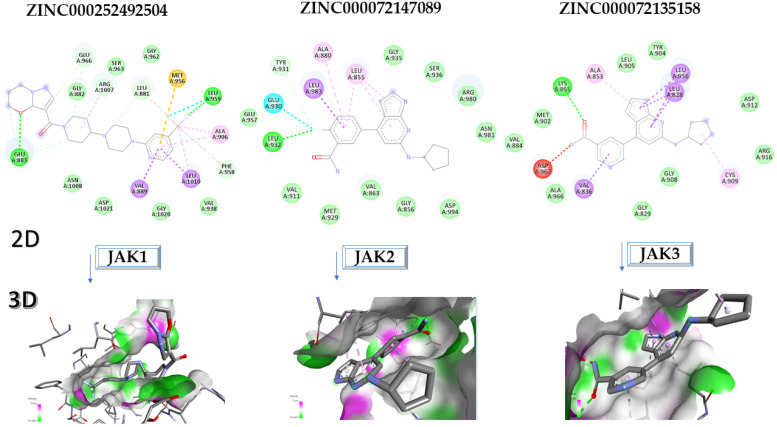
Interactions between amino acids of JAK1, JAK2, and JAK3, and the most promising compounds tested in 2D and 3D formats.

Figure 8 presents the in-depth 2D and 3D interactions of the most promising, newly identified ligands for their high affinity for JAKs.

### 2.5. In Silico Evaluation of the Pharmacokinetic Profile of Newly Identified Compounds

Evaluations of the pharmacokinetic profile of the newly discovered compounds were performed in silico using the SwissADME (http://www.swissadme.ch/, accessed on 9 April 2023) online platform. Important parameters were identified that can facilitate the design of a framework for future possible uses of the three newly identified compounds.

Table 7 shows the results with pharmacokinetic implications obtained after processing the data introduced into the platform.

The molecular weights of the three compounds place them in the category of small molecules, which tend to be absorbed more easily and eliminated more quickly. The properties of the three newly identified compounds comply with all of Lipinski’s principles for predicting the oral bioavailability of a small drug molecule: molecular weight less than 500 g/mol, maximum 10 H-bond acceptors, maximum 5 H-bond donors, LogP (octanol–water partition coefficient) less than 5, and number of rotatable bonds less than 5. Small, lipophilic, or hydrophobic molecules with a limited number of hydrogen bond donors and acceptors are more likely to achieve good oral bioavailability because they can easily pass through the cellular membrane in the intestines and reach the bloodstream, according to the Lipinski principles [42]. 

The action on cytochrome P450 results from their inductive or inhibitory action on some of the most important isoforms. Future research should confirm these characteristics because such actions may involve in vivo interactions or reduce or exacerbate the pharmacological effect [43]. 

Interpretation of pharmacokinetic data includes newly discovered compounds in the category of small drug molecules with the potential to be administered orally and to offer the advantages that come with this route of administration.

SwissADME employs computational models and algorithms to estimate crucial ADME parameters, including solubility, lipophilicity, permeability, and pharmacokinetics. Utilizing molecular descriptors and machine learning techniques, it provides estimates based on the chemical structure of the molecule. Despite the fact that in silico methods for assessing drug ADME properties can be beneficial in the early stages of drug development, it is essential to emphasize that they do not replace in vivo investigations; rather, these studies aim to provide a cost-effective and time-efficient method for analyzing the pharmacokinetic properties of small molecules [44].

Virtual screening, pharmacokinetic studies, and molecular docking are effective computational technologies that are currently applied in the research and development of new drugs. Using precise molecular simulation models and analyses to predict the binding affinity of potential active molecules to their target proteins, emphasizing knowledge of drug–protein interactions, and enhancing the ability to develop and optimize new molecules are among the most relevant advantages of in silico studies. They enable the quick assessment of a large number of potential active molecule candidates while minimizing costs and time compared to traditional wet-lab experiments [45,46,47].

The advantages produced by these techniques were also exploited in the present study, in which in silico studies on the interaction with three JAK isoforms were conducted, providing, through a comprehensive approach, three compounds with potential input for extensive laboratory studies regarding RA for accurately determining their safety and efficacy profile.

The present study has a few limitations related to the types of evaluations conducted. Although useful insights on compound–target interactions have been obtained through molecular docking simulations, these findings still require experimental confirmation. The experimental validation method can be time- and money-consuming, and the outcomes might not always agree with the simulations’ predictions. Molecular docking evaluations focus on a single static interaction between the compound and its target protein, whereas proteins are highly dynamic in the biological milieu and can significantly alter their conformation during compound binding. Numerous approximations have been made in molecular docking simulations, including the exclusion of other proteins from the cellular environment and the neglect of solvent effects. These assumptions may reduce the simulations’ accuracy and result in inaccurate estimations.

The presence of fluorine atoms in the structure of some compounds identified in ligand-based virtual screening analyses may present a number of limitations due to their pronounced electronegative character and the likelihood of forming very strong bonds with any protein, hence the need for verification of the parent compound used as a ligand as well as experimental optimization and validation in future studies. Although fluorine can enhance chemical stability, the introduction of fluorine atoms can also impact the metabolic fate and toxicity of a compound. Some fluorinated compounds may have longer half-lives or different metabolic profiles, which could impact their safety and suitability as drug candidates. Furthermore, the presence of fluorine in a molecule may complicate the synthesis process, as fluorination reactions may be more challenging and require specialized reagents and conditions.

These limitations enable improvements in the study design by opening research directions through the application of network pharmacology and molecular dynamics, which address the dynamic nature of proteins and possible interactions, the effect of solvents and ions, as well as simulating the presence of other cellular structures that may influence the interaction.

## 3. Materials and Methods

### 3.1. Identification and Selection of Target Proteins

Clinical evidence demonstrated that JAKs play significant roles in specific pathogenic RA pathways, and research on JAKis resulted in their approval as RA treatments.

The complexity of the role of JAKs in the pathophysiology of RA, as well as the promising results obtained from the use of JAKis, renders the above-mentioned proteins highly essential in the overall management of RA and makes them suitable for in silico studies. Therefore, JAK1, JAK2, and JAK3 with PDB (https://www.rcsb.org/, accessed on 25 April 2023) IDs 3EYG, 6VNE, and 5TTV were selected as target proteins for the present molecular docking study. For all three target JAKs, ligands selected based on affinity for these structures have been docked.

### 3.2. Ligand Preparation

A total of 8 chemical compounds—5 approved (tofacitinib, baricitinib, peficitinib, upadacitinib, and filgotinib) and 3 in studies (itacitinib, ruxolitinib, and decernotinib)—were selected and processed in Simulation Description Format (SDF) using the PubChem database (https://pubchem.ncbi.nlm.nih.gov/, accessed on 17 April 2023). Since .SDF formatting is not compatible with the molecular docking software used (AutoDock Tools 1.5.7, https://vina.scripps.edu/, accessed on 20 March 2023), these files were converted to the specific AutoDock Tools 1.5.7 format (Protein Data Bank, Partial Charge (Q), and Atom Type (T) format). Furthermore, ligands were imported into the AutoDockTools software using the ligand input function to add charges and determine rotable bonds [48].

### 3.3. Protein Preparation

The data required for the JAK1 (3EYG), JAK2 (6VNE), and JAK3 (5TTV) studies were obtained from the Research Collaboratory for Structural Bioinformatics PDB (https://www.rcsb.org/, accessed on 6 April 2023) online platform.

To perform the molecular docking assay, a ligand-free and co-crystallized water-free protein is required, and Molegro Molecular Viewer 7.0 software (https://molexus.io/molegro-molecular-viewer/, accessed on 14 April 2023) has been used in this regard. Furthermore, polar hydrogens and Gasteiger charges were added using AutoDockTools 1.5.7 [48].

### 3.4. Molecular Docking Analysis

Molecular docking studies were performed using AutoDockTools 1.5.7, and 2D and 3D images were generated using Discovery Studio Visualizer 4.5 (https://www.3ds.com/products-services/biovia/products/molecular-modeling-simulation/biovia-discovery-studio/visualization/, accessed on 20 April 2023). Furthermore, Molegro Molecular Viewer 7.0 software (http://molexus.io/molegro-molecular-viewer/, accessed on 22 April 2023) was used in the analysis of the protein preparation workflow and Open Babel GUI 3.0.1 in the ligand preparation process [49]. The docking procedure was validated by re-docking the compound with which the protein co-crystallizes. The grid box size was set to 60 × 60 × 60 Å. As an approach for validating the docking process, the re-docking of the native ligand (i.e., the ligand molecule that is co-crystallized with the target protein) back into its own protein binding pocket was performed via the previously described molecular docking method. The docking algorithm aims to reproduce the experimentally observed binding conformation of the native ligand. By comparing the docked pose to the crystallographic pose, the docking algorithm’s accuracy can be determined. Through the RMSD, the similarity between the atomic coordinates of the two poses is determined. In the setting of docking evaluation, the RMSD is obtained relative to the native ligand to assess the degree to which the predicted pose corresponds to the crystallographic pose. In the field of computational molecular docking, algorithms that generate poses with RMSD values below 2 Å, with RMSD calculated relative to the native ligand, are regarded as reliable and valid. A lower RMSD value suggests a better match between the predicted and experimental poses, indicating that the docking algorithm is more accurate [50]. The RMSD value calculated with AutodockTools 1.5.7. software [48] falls within the literature-accepted range (<2 Å) for all analyses performed in this study. 

### 3.5. Virtual Screening

For finding new compounds with potential in the treatment of RA, the chemical structure of the compounds that showed the highest affinity for JAK1, JAK2, and JAK3 following docking studies was used to identify compounds with similar chemical structure and therapeutic potential using the online platform SwissSimilarity (http://www.swisssimilarity.ch/, accessed on 15 April 2023). The ligands were downloaded in .SDF format from the ZINC database, converted with OpenBabelGUI 3.0.1 (https://openbabel.org/docs/current/GUI/GUI.html/, accessed on 20 March 2023) [49] into a format compatible with AutoDock Vina 1.2.0 (https://vina.scripps.edu/, accessed on 20 March 2023), and with the Autodock Vina 1.2.0 software the necessary charges were added, and the rotatable bonds were determined [48].

### 3.6. Pharmacokinetic Profile Predictions

Predictions of the pharmacokinetic profile of compounds that were identified as most promising from molecular docking and virtual screening analyses were performed using the SwissADME online tool (http://www.swissadme.ch/, accessed on 15 April 2023).

## 4. Conclusions

JAKis have the potential to reduce the progression of joint deterioration and improve patient outcomes by targeting the inflammation underlying RA, which has led to the approval of five compounds in therapy. However, rheumatological medical practice suggests incomplete control of RA in patients, which determines the need for further studies on identifying new compounds with biological activity.

Molecular docking studies continue to be an important tool in the discovery and development of new drugs for RA and other inflammatory diseases. They provide important insights into the interactions between small molecules and protein targets, helping to identify the most promising compounds for further development.

Through the process of virtual screening, starting with the analysis of affinities for the target proteins of some JAKis approved in RA pharmacotherapy or present in advanced phases of research, the present in silico study has identified three new promising compounds (ZINC252492504, ZINC72147089, and ZINC72135158) not used in medical practice. These compounds may represent a medium-to-long-term solution if these results are coupled with further research on the extensive study of the efficacy and safety profiles of these molecules.

## Figures and Tables

**Table 1:** Docking scores of the compounds investigated.

Ligands	Binding Affinity (kcal/mol)	Structure
Baricitinib	−9.0	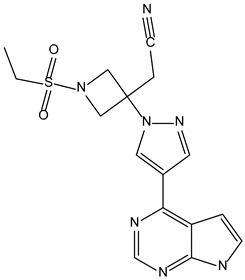
Upadacitinib	−6.4	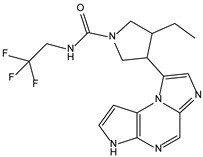
Ruxolitinib	−9.0	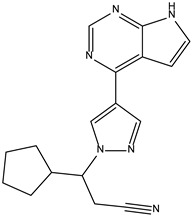
Itacitinib	−9.7	
Peficitinib	−6.5	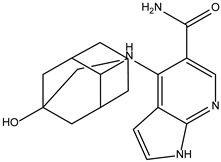
Filgotinib	−9.1	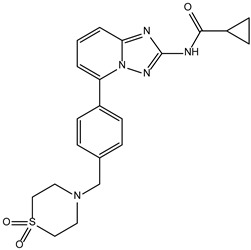

**Table 2:** Docking scores of the investigated compounds.

Ligands	Binding Affinity (kcal/mol)
Baricitinib	−8.3
Ruxolitinib	−8.5
Peficitinib	−9.5

**Table 3:** Docking scores of the investigated compounds.

Ligands	Binding Affinity (kcal/mol)
Decernotinib	−8.5
Tofacitinibului	−8.4
Peficitinib	−9.5

**Table 4:** The first five most promising compounds with action on JAK1 identified in the ZINC database.

Compound	Structure	Binding Affinity (kcal/mol)	Similarity Score
ZINC000252492504	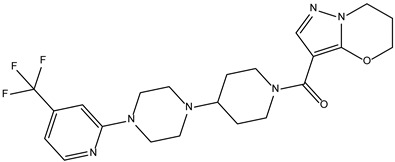	−9.0	0.303
ZINC000272517982	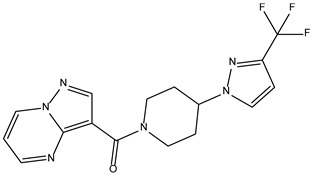	−8.8	0.301
ZINC000584884024	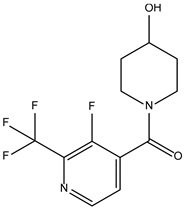	−7.8	0.442
ZINC000530112879	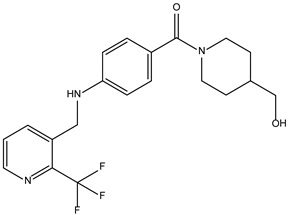	−7.7	0.304
ZINC000013974879	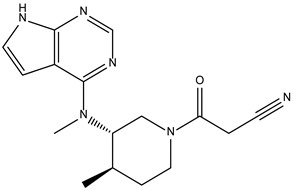	−7.4	0.337

**Table 5:** The five most promising compounds with potential action on JAK2 identified in ZINC.

Compound	Structure	Binding Affinity (kcal/mol)	Similarity Score
ZINC000072147089	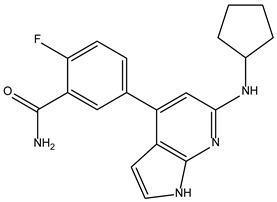	−8.6	0.325
ZINC000239174350	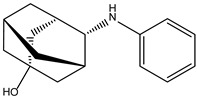	−8.5	0.400
ZINC000016384670	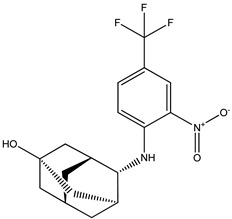	−8.5	0.338
ZINC000072135158	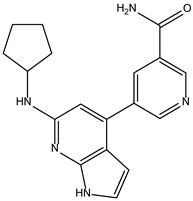	−8.5	0.313
ZINC000263818685	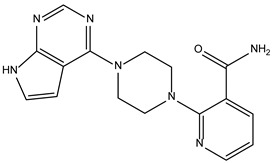	−8.3	0.342

**Table 6:** The first five most promising compounds with action on JAK3 identified in ZINC.

Compound	Structure	Binding Affinity (kcal/mol)	Similarity Score
ZINC000072135158	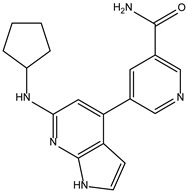	−8.6	0.313
ZINC000072147089	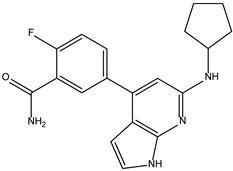	−8.5	0.325
ZINC000072149208	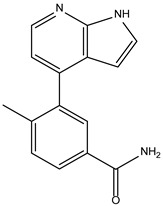	−8.5	0.319
ZINC000239174350	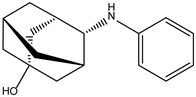	−8.4	0.400
ZINC000214499182	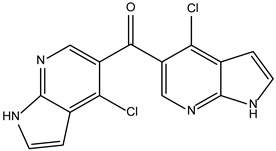	−8.0	0.333

**Table 7:** Pharmacokinetic features of the newly identified compounds.

Characteristics	ZINC000252492504 (JAK1)	ZINC000072147089 (JAK2)	ZINC000072135158 (JAK3)
Formula	C_22_H_27_F_3_N_6_O_2_	C_19_H_19_FN_4_O	C_18_H_19_N_5_O
Molecular weight	464.48 g/mol	338.38 g/mol	321.38 g/mol
Num. rotatable bonds	5	4	4
Num. H-bond acceptors	8	3	3
Num. H-bond donors	0	3	3
Log P	3.37	2.34	1.89
Gastrointestinal absorption	High	High	High
CYP2C19 inhibitor	Yes	No	No
CYP2C9 inhibitor	Yes	No	No
CYP2D6 inhibitor	Yes	Yes	Yes
CYP3A4 inhibitor	Yes	Yes	Yes
Lipinski	Yes; 0 violation	Yes; 0 violation	Yes; 0 violation

## Data Availability

Data presented in this paper are supported by the inserted references and by the results obtained through experimental part.
